# Increasing incidence and improving survival of oral tongue squamous cell carcinoma

**DOI:** 10.1038/s41598-020-64748-0

**Published:** 2020-05-12

**Authors:** Yi-Jun Kim, Jin Ho Kim

**Affiliations:** 1 Institute of Convergence Medicine, Ewha Womans University Mokdong Hospital, Seoul, Republic of Korea; 2 Department of Radiation Oncology, Ewha Womans University College of Medicine, Seoul, Republic of Korea; 3 Department of Radiation Oncology, Seoul National University College of Medicine, Seoul, Republic of Korea

**Keywords:** Cancer epidemiology, Oral cancer

## Abstract

We evaluated changes in incidence, relative survival (RS), and conditional survival (CS) of head and neck squamous cell carcinoma (HNSCC), focusing on oral tongue squamous cell carcinoma (OTSCC). Data of 74 680 HNSCC patients from 1976 to 2015 were obtained from the Surveillance, Epidemiology, and End Results database. Five anatomical sites and their subsites were analyzed. Annual percent change (APC) of incidence was calculated. RS and CS were compared across the four decades. Adjusted hazard ratios (aHRs) of RS were evaluated using multivariate regression. OTSCC incidence decreased from 1976 (APC = −0.76, *P* < 0.05) but has increased since 1999 (APC = 2.36, *P* < 0.05). During 2006–2015, the 5-year CS exceeded 90% only for OTSCC and oropharyngeal squamous cell carcinoma (OPSCC). RS improved in OTSCC (aHR = 0.697, 95% confidence interval [CI] 0.642–0.757, *P* < 0.001) and OPSCC (aHR = 0.669, 95% CI 0.633–0.706, *P* < 0.001) during the last two decades. For both OTSCC and OPSCC, improved survival was observed regardless of treatment. Incidence and survival remained unchanged for nasopharyngeal, hypopharyngeal, and laryngeal cancers during this period. In conclusion, OTSCC incidence has been increasing since the 2000s, with improving prognosis irrespective of treatment. Given its similarity to OPSCC, OTSCC may represent an emerging HNSCC, warranting further research and clinical recognition.

## Introduction

In 2019, there were about 65,410 new cases of head and neck cancers in the United States, which accounted for about 3.7% of new cancers^1^. Changes in the survival rate of head and neck cancer depend on many factors, including the epidemiological trends of the diseases and therapeutic developments^2,3^.

To date, we have accumulated a body of knowledge, regarding head and neck cancers in a number of organs. First, smoking has been a significant cause of head and neck squamous cell carcinoma (HNSCC)^4^. Over the last few decades, the incidence of oral cavity cancer has decreased as smoking has decreased^5^. Recently, however, several studies have found that, among the oral cavity subsite cancers, the incidence of oral tongue squamous cell carcinoma (OTSCC, anterior 2/3 of tongue), has increased^6–8^. Second, the incidence of human papillomavirus (HPV)-related oropharyngeal squamous cell carcinoma (OPSCC, tonsil, base of tongue, soft palate) has gradually increased over past decades and is now the major type of head and neck cancer^9^. This cancer is considered to be a sexually transmitted disease. It has shown a good prognosis and has been met with better treatment responses than HPV-unrelated OPSCC^10–13^. Third, survival rates are reported to have increased, in varying degrees on a scale of decades, for nasopharynx, hypopharynx, and laryngeal cancers^14–16^.

However, we are still lacking knowledge regarding a number of the characteristics of head and neck cancers. It is not known whether oral cavity cancer, the incidence of which has recently been rising, has also seen increases in survival rates, as has OPSCC. In addition, unlike clinical changes in oropharynx cancer, those in nasopharynx, hypopharynx, and larynx cancers have rarely been investigated by long-term observational studies. Some researchers have studied the possibility that HPV-related head and neck cancers may occur in non-oropharyngeal sites and have aimed to determine the impact of HPV infection on related outcomes^17,18^. However, it is not clear whether cancers in these organs have changed in incidence and survival rates, as has been the case in oropharyngeal cancer.

Identifying the time-sequential changes in incidence and survival rates, by organ site, of head and neck cancers will provide important evidence regarding their epidemiological changes. The current study determined the annual percent change (APC) of incidence, relative survival (RS), and conditional survival (CS) in HNSCC patients who were diagnosed during the four consecutive calendar decades from 1976 to 2015 using the Surveillance, Epidemiology, and End Results (SEER) database.

## Results

### Patient characteristics and Annual percent change (APC)

A total of 74 680 patients with HNSCC were included in the study (Supplementary Fig. S1). Patient characteristics according to the four calendar decades are presented in Table 1. Treatment characteristics and tumor extent are described in Tables 2 and 3. The highest incidence of head and neck cancer was found in patients who are aged ≥50 years, white, and male sex. The time-sequential changes of age, sex, and race of head and neck cancer patients were not prominent. The number of OPSCC showed gradual increments over the four decades. An increase in the number of OTSCC cases contributed to a modest increase in oral cavity cancer incidence. The number of hypopharyngeal and laryngeal cancers apparently decreased during the same period. Usage of chemotherapy and radiotherapy gradually increased. There was no remarkable changes in the extent of disease and tumor grade over time.

**Table 1:** Characteristics of HNSCC patients according to the diagnostic period of the SEER database.

Characteristics	1976–1985		1986–1995		1996–2005		2006–2015	
	n = 17 298		n = 16 821		n = 17 343		n = 23 218	
	no.	(%)	no.	(%)	no.	(%)	no.	(%)
**Age**								
<50	2 107	(12.2)	2 581	(15.3)	3 239	(18.7)	3 209	(13.8)
50–59	4 793	(27.7)	3 766	(22.4)	4 760	(27.4)	7 477	(32.2)
60–69	5 894	(34.1)	5 280	(31.4)	4 192	(24.2)	6 939	(29.9)
>70	4 504	(26.0)	5 194	(30.9)	5 152	(29.7)	5 593	(24.1)
**Sex**								
Male	12 647	(73.1)	12 180	(72.4)	12 661	(73.0)	17 753	(76.5)
Female	4 651	(26.9)	4 641	(27.6)	4 682	(27.0)	5 465	(23.5)
**Race**								
NH-White	14 082	(81.4)	13 042	(77.5)	12 943	(74.6)	17 592	(75.8)
NH-Black	2 035	(11.8)	2 187	(13.0)	2 212	(12.8)	2 402	(10.3)
NH-AIAN	24	(0.1)	65	(0.4)	96	(0.6)	148	(0.6)
NH-API	698	(4.0)	904	(5.4)	1 254	(7.2)	1 627	(7.0)
NH-unknown race	37	(0.2)	29	(0.2)	63	(0.4)	165	(0.7)
Hispanic	422	(2.4)	594	(3.5)	775	(4.5)	1 284	(5.5)
**Site**								
Oral cavity	5 051	(29.2)	4 746	(28.2)	4 703	(27.1)	6 014	(25.9)
Tongue, oral	1 711	(9.9)	1 840	(10.9)	2 132	(12.3)	3 277	(14.1)
Others	3 340	(19.3)	2 906	(17.3)	2 571	(14.8)	2 737	(11.8)
Oropharynx	3 661	(21.2)	3 753	(22.3)	5 176	(29.8)	9 266	(39.9)
Tonsil	1 497	(8.7)	1 572	(9.3)	2 368	(13.7)	4 457	(19.2)
Base of tongue	1 243	(7.2)	1 349	(8.0)	1 981	(11.4)	3 693	(15.9)
Soft palate	501	(2.9)	421	(2.5)	347	(2.0)	300	(1.3)
Others	420	(2.4)	411	(2.4)	480	(2.8)	816	(3.5)
Nasopharynx	806	(4.7)	843	(5.0)	873	(5.0)	924	(4.0)
Hypopharynx	1 599	(9.2)	1 530	(9.1)	1 201	(6.9)	1 031	(4.4)
Larynx	6 181	(35.7)	5 949	(35.4)	5 390	(31.1)	5 983	(25.8)
Supraglottis	2 025	(11.7)	1 953	(11.6)	1 958	(11.3)	2 151	(9.3)
Glottis	3 275	(18.9)	3 208	(19.1)	2 799	(16.1)	3 173	(13.7)
Others	881	(5.1)	788	(4.7)	633	(3.6)	659	(2.8)
**Extent of disease**								
Local	6 068	(35.1)	5 611	(33.4)	5 450	(31.4)	7 708	(33.2)
Regional	7 889	(45.6)	8 336	(49.6)	9 349	(53.9)	11 529	(49.7)
Distant	2 238	(12.9)	1 802	(10.7)	1 927	(11.1)	3 290	(14.2)
Unknown/blank(s)	1 103	(6.4)	1 072	(6.4)	617	(3.6)	691	(3.0)
**Grade**								
Well differentiated	3 509	(20.3)	2 784	(16.6)	2 060	(11.9)	2 622	(11.3)
Moderately differentiated	5 886	(34.0)	6 729	(40.0)	7 254	(41.8)	8 932	(38.5)
Poorly differentiated	3 286	(19.0)	4 031	(24.0)	4 671	(26.9)	6 141	(26.4)
Undifferentiated, anaplastic	207	(1.2)	202	(1.2)	288	(1.7)	302	(1.3)
Unknown	4 410	(25.5)	3 075	(18.3)	3 070	(17.7)	5 221	(22.5)
**Surgery**								
No/unknown	7 935	(45.9)	7 311	(43.5)	7 997	(46.1)	12 578	(54.2)
Yes	9 363	(54.1)	9 510	(56.5)	9 346	(53.9)	10 640	(45.8)
**Chemotherapy**								
No/unknown	15 445	(89.3)	14 069	(83.6)	11 867	(68.4)	11 475	(49.4)
Yes	1 853	(10.7)	2 752	(16.4)	5 476	(31.6)	11 743	(50.6)
**Radiotherapy**								
No/unknown	6 108	(35.3)	5 338	(31.7)	5 028	(29.0)	6 654	(28.7)
Yes	11 190	(64.7)	11 483	(68.3)	12 315	(71.0)	16 564	(71.3)

Abbreviations: HNSCC, head and neck squamous cell carcinoma; SEER, Surveillance, Epidemiology, and End Results; NH, non-Hispanic; AIAN, American Indian/Alaska Native; API, Asian or Pacific Islander.

**Table 2:** Treatment according to the subsite and diagnostic period in HNSCC of the SEER database.

Characteristics	1976–1985		1986–1995		1996–2005		2006–2015	
	n = 17 298		n = 16 821		n = 17 343		n = 23 218	
	no.	(%)	no.	(%)	no.	(%)	no.	(%)
**Oral cavity**, **anterior tongue**								
Surgery								
No/unknown	515	(30.1)	443	(24.1)	400	(18.8)	524	(16.0)
Yes	1 196	(69.9)	1 397	(75.9)	1 732	(81.2)	2 753	(84.0)
Chemotherapy								
No/unknown	1 539	(89.9)	1 644	(89.3)	1 865	(87.5)	2 551	(77.8)
Yes	172	(10.1)	196	(10.7)	267	(12.5)	726	(22.2)
Radiotherapy								
No/unknown	927	(54.2)	1 048	(57.0)	1 234	(57.9)	2 049	(62.5)
Yes	784	(45.8)	792	(43.0)	898	(42.1)	1 228	(37.5)
**Oral cavity**, **others**								
Surgery								
No/unknown	1 082	(32.4)	797	(27.4)	649	(25.2)	649	(23.7)
Yes	2 258	(67.6)	2 109	(72.6)	1 922	(74.8)	2 088	(76.3)
Chemotherapy								
No/unknown	3 028	(90.7)	2 584	(88.9)	2 235	(86.9)	2 066	(75.5)
Yes	312	(9.3)	322	(11.1)	336	(13.1)	671	(24.5)
Radiotherapy								
No/unknown	1 724	(51.6)	1 455	(50.1)	1 285	(50.0)	1 499	(54.8)
Yes	1 616	(48.4)	1 451	(49.9)	1 286	(50.0)	1 238	(45.2)
**Oropharynx**								
Surgery								
No/unknown	2 187	(59.7)	1 996	(53.2)	2 676	(51.7)	5 996	(64.7)
Yes	1 474	(40.3)	1 757	(46.8)	2 500	(48.3)	3 270	(35.3)
Chemotherapy								
No/unknown	3 018	(82.4)	2 822	(75.2)	2 802	(54.1)	2 564	(27.7)
Yes	643	(17.6)	931	(24.8)	2 374	(45.9)	6 702	(72.3)
Radiotherapy								
No/unknown	891	(24.3)	883	(23.5)	985	(19.0)	1 446	(15.6)
Yes	2 770	(75.7)	2 870	(76.5)	4 191	(81.0)	7 820	(84.4)
**Nasopharynx**								
Surgery								
No/unknown	673	(83.5)	719	(85.3)	682	(78.1)	837	(90.6)
Yes	133	(16.5)	124	(14.7)	191	(21.9)	87	(9.4)
Chemotherapy								
No/unknown	674	(83.6)	557	(66.1)	216	(24.7)	160	(17.3)
Yes	132	(16.4)	286	(33.9)	657	(75.3)	764	(82.7)
Radiotherapy								
No/unknown	111	(13.8)	102	(12.1)	89	(10.2)	131	(14.2)
Yes	695	(86.2)	741	(87.9)	784	(89.8)	793	(85.8)
**Hypopharynx**								
Surgery								
No/unknown	798	(49.9)	800	(52.3)	752	(62.6)	851	(82.5)
Yes	801	(50.1)	730	(47.7)	449	(37.4)	180	(17.5)
Chemotherapy								
No/unknown	1 316	(82.3)	1 085	(70.9)	636	(53.0)	290	(28.1)
Yes	283	(17.7)	445	(29.1)	565	(47.0)	741	(71.9)
Radiotherapy								
No/unknown	404	(25.3)	339	(22.2)	265	(22.1)	211	(20.5)
Yes	1 195	(74.7)	1 191	(77.8)	936	(77.9)	820	(79.5)
**Larynx**								
Surgery								
No/unknown	2 680	(43.4)	2 556	(43.0)	2 838	(52.7)	3 721	(62.2)
Yes	3 501	(56.6)	3 393	(57.0)	2 552	(47.3)	2 262	(37.8)
Chemotherapy								
No/unknown	5 870	(95.0)	5 377	(90.4)	4 113	(76.3)	3 844	(64.2)
Yes	311	(5.0)	572	(9.6)	1 277	(23.7)	2 139	(35.8)
Radiotherapy								
No/unknown	2 051	(33.2)	1 511	(25.4)	1 170	(21.7)	1 318	(22.0)
Yes	4 130	(66.8)	4 438	(74.6)	4 220	(78.3)	4 665	(78.0)

Abbreviations: HNSCC, head and neck squamous cell carcinoma; SEER, Surveillance, Epidemiology, and End Results.

**Table 3:** Extent of disease according to the subsite and diagnostic period in HNSCC of the SEER database.

Extent of disease	1976–1985		1986–1995		1996–2005		2006–2015	
	n = 17 298		n = 16 821		n = 17 343		n = 23 218	
	no.	(%)	no.	(%)	no.	(%)	no.	(%)
**Oral cavity**, **anterior tongue**								
Local	879	(51.4)	977	(53.1)	1190	(55.8)	1828	(55.8)
Regional	573	(33.5)	524	(28.5)	609	(28.6)	976	(29.8)
Distant	127	(7.4)	186	(10.1)	244	(11.4)	365	(11.1)
Unknown	132	(7.7)	153	(8.3)	89	(4.2)	108	(3.3)
**Oral cavity**, **others**								
Local	1006	(30.1)	831	(28.6)	751	(29.2)	870	(31.8)
Regional	1738	(52.0)	1562	(53.8)	1508	(58.7)	1494	(54.6)
Distant	288	(8.6)	223	(7.7)	164	(6.4)	237	(8.7)
Unknown	308	(9.2)	290	(10.0)	148	(5.8)	136	(5.0)
**Oropharynx**								
Local	683	(18.7)	566	(15.1)	580	(11.2)	772	(8.3)
Regional	1989	(54.3)	2405	(64.1)	3679	(71.1)	6401	(69.1)
Distant	754	(20.6)	561	(14.9)	779	(15.1)	1957	(21.1)
Unknown	235	(6.4)	221	(5.9)	138	(2.7)	136	(1.5)
**Nasopharynx**								
Local	142	(17.6)	97	(11.5)	115	(13.2)	162	(17.5)
Regional	398	(49.4)	542	(64.3)	638	(73.1)	619	(67.0)
Distant	193	(23.9)	143	(17.0)	88	(10.1)	98	(10.6)
Unknown	73	(9.1)	61	(7.2)	32	(3.7)	45	(4.9)
**Hypopharynx**								
Local	226	(14.1)	149	(9.7)	98	(8.2)	95	(9.2)
Regional	920	(57.5)	1060	(69.3)	803	(66.9)	493	(47.8)
Distant	376	(23.5)	261	(17.1)	269	(22.4)	427	(41.4)
Unknown	77	(4.8)	60	(3.9)	31	(2.6)	16	(1.6)
**Larynx**								
Local	3132	(50.7)	2991	(50.3)	2716	(50.4)	3981	(66.5)
Regional	2271	(36.7)	2243	(37.7)	2112	(39.2)	1546	(25.8)
Distant	500	(8.1)	428	(7.2)	383	(7.1)	206	(3.4)
Unknown	278	(4.5)	287	(4.8)	179	(3.3)	250	(4.2)

Abbreviations: HNSCC, head and neck squamous cell carcinoma; SEER, Surveillance, Epidemiology, and End Results.

**Figure 1:**
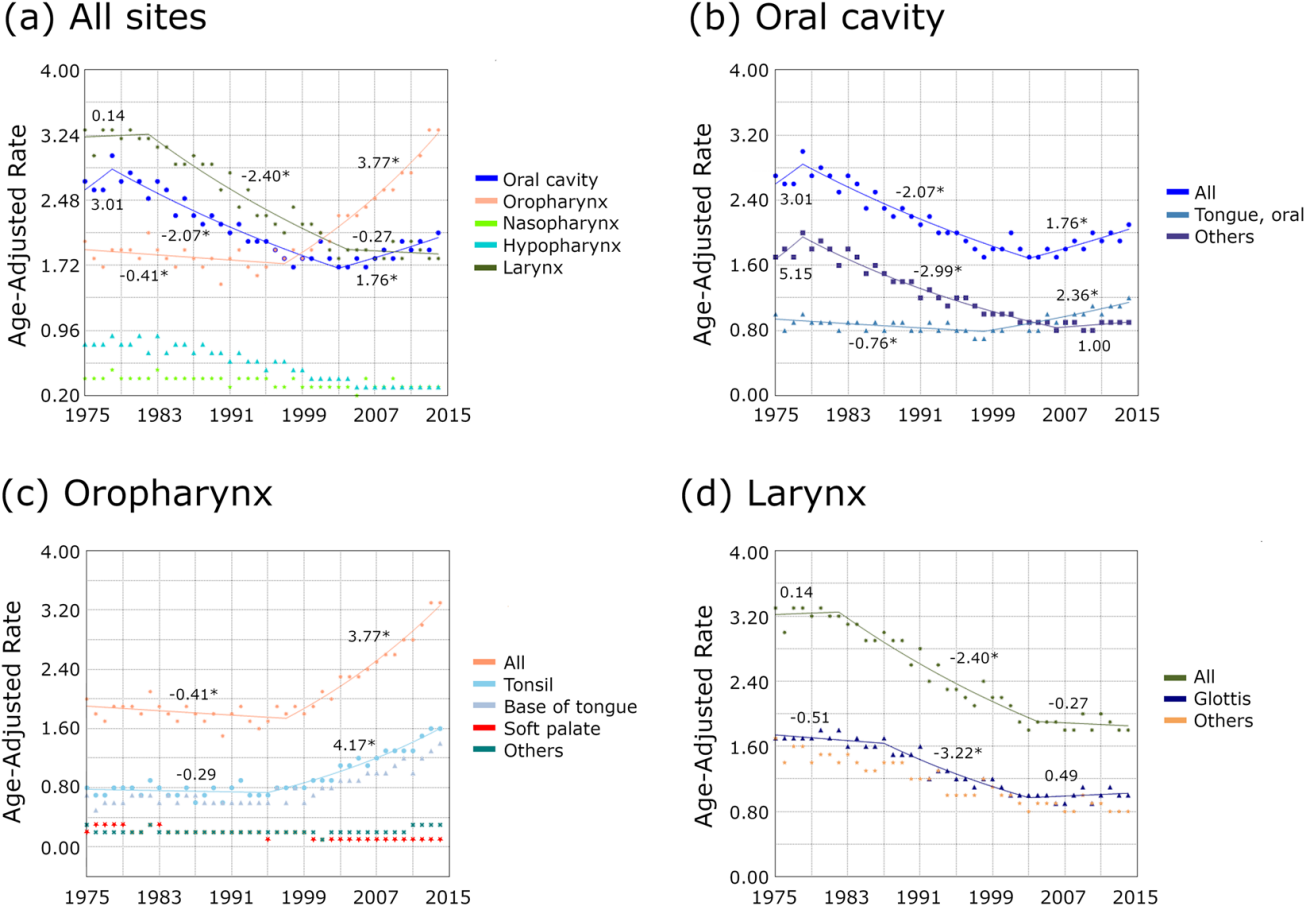
Annual percent change of head and neck squamous cell carcinoma from 1976 to 2015. (**a**) All five anatomic sites, (**b**) oral cavity, (**c**) oropharynx, and (**d**) larynx. Asterisk indicates that the APC is significantly different from zero at the alpha = 0.05 level. APC, Annual percent change.

Age-adjusted incidence of HNSCC was analyzed according to the five anatomical sites (Fig. 1). The incidence of oral cavity and laryngeal cancers significantly decreased and that of nasopharyngeal and hypopharyngeal cancers remained steady. The incidence of OPSCC has been increasing significantly since 1998 (Fig. 1a). For oral cavity, oropharyngeal, and laryngeal cancers, subgroup analysis was performed according to their subsites (Fig. 1b to d). The incidence of OTSCC has significantly increased from 1999 (APC = 2.36; *P* < 0.05) whereas that of other oral cavity subsite cancers significantly decreased between 1979 and 2007 (APC = −2.99; *P* < 0.05) (Fig. 1b). Among OPSCCs, the incidence of tonsillar cancer has significantly increased since 1996 (APC = 4.17; *P* < 0.05) (Fig. 1c). Incidence of glottis cancer (a subsite of laryngeal cancer) significantly decreased from 1988 to 2004 (APC = −3.22, *P* < 0.05) (Fig. 1d). Among HNSCCs, only the incidence rates of OTSCC and OPSCC demonstrated an overall increase since the 2000s.

### RS and CS from 1976 to 2015

RS and CS were computed and compared across the four consecutive calendar decades from 1976 to 2015.

**Figure 2:**
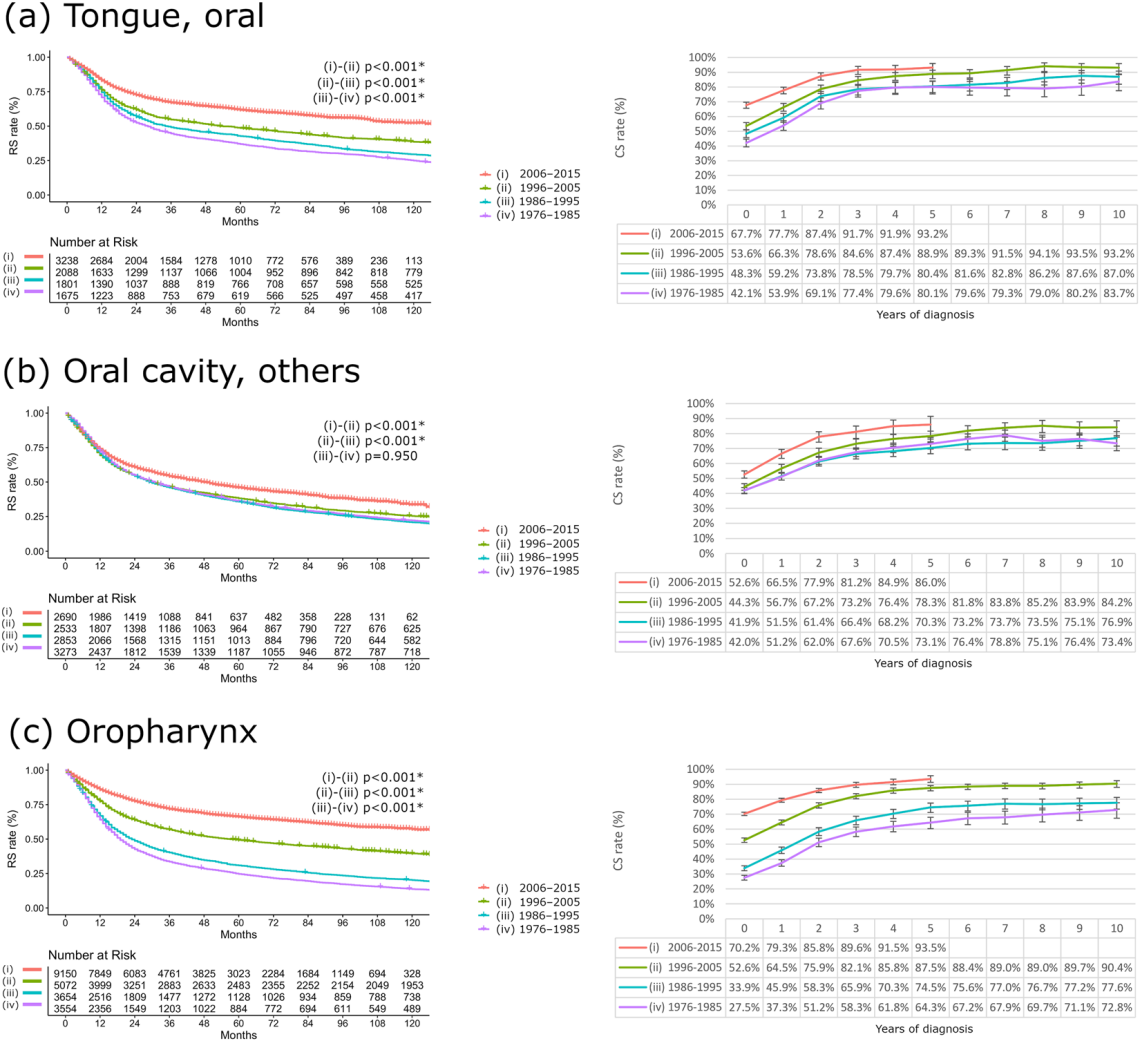
Relative survival (RS) and 5-year conditional survival (CS) estimates for head and neck squamous cell carcinoma according to each anatomical site (oral tongue, oral cavity others, and oropharynx) during the four calendar periods. For each calendar decade, a series of CSs was provided at the consecutive post-diagnosis years. Asterisks represent statistical significance after adjustment by the Bonferroni correction (P < 0.05/6).

During this period, the improvement in RS differed among cancer sites (Fig. 2 and Supplementary Fig. S2). Across the four calendar decades, the absolute increases in 5-year RS were 17.9% for oral cavity cancers (curve not shown), 41.5% for oropharyngeal cancer, 21.6% for nasopharyngeal cancer, 16.5% for hypopharyngeal cancer, and 5.2% for laryngeal cancer. Subgroup analysis revealed that OTSCC showed greater improvement in survival than other oral cavity subsite cancers during the four decades (from 37.0% in 1976–1985 to 61.7% in 2006–2015). The absolute 5-year RS increments were 24.7% vs. 9.5% for OTSCC vs. other oral cavity cancers. Apart from OPSCC, OTSCC showed the greatest absolute increase in survival. It is notable that survival increments for both cancers were observed mainly during the last two decades (Fig. 2a,c). RS in each clinical feature groups were analyzed according to the organ sites and the diagnostic periods (Supplementary Table S1-S6). Among the organ sites, OTSCC and OPSCC showed remarkably improved survival irrespective of extent of disease, tumor grade, and treatments.

In the most recent calendar decade (2006–2015), the 5-year CS rates were 90.8% (not shown), 93.5%, 84.9%, 77.2%, and 85.7% for oral cavity, oropharyngeal, nasopharyngeal, hypopharyngeal, and laryngeal cancers, respectively (Fig. 2 and Supplementary Fig. S2). Subgroup analysis revealed that 5-year CS was better in OTSCC than in other oral cavity sites (93.2% vs. 86.0%). As of 2015, only OPSCC and OTSCC showed 5-year CS rates > 90% (93.2% and 93.5%, respectively).

### Multivariate analysis

**Figure 3:**
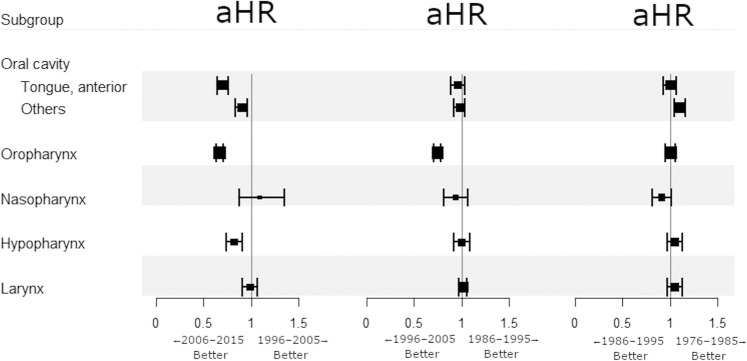
Effect of consecutive calendar decades on survival within anatomical sites variables. Adjustment for age, sex, race, cancer site, extent of disease, grade, surgery, chemotherapy, and radiotherapy was performed for each subgroup (each row). aHR, adjusted hazard ratio.

**Figure 4:**
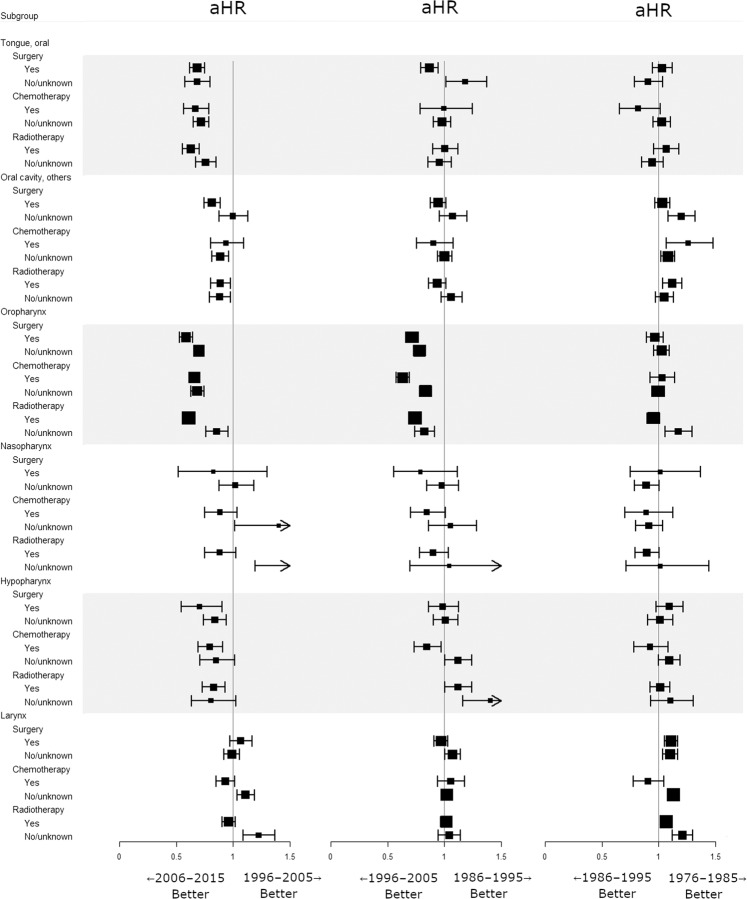
Effect of consecutive calendar decades on survival within treatment variables stratified by primary sites. Multivariate adjustment was done to obtain each aHR as in Fig. 3. aHR, adjusted hazard ratio.

**Figure 5:**
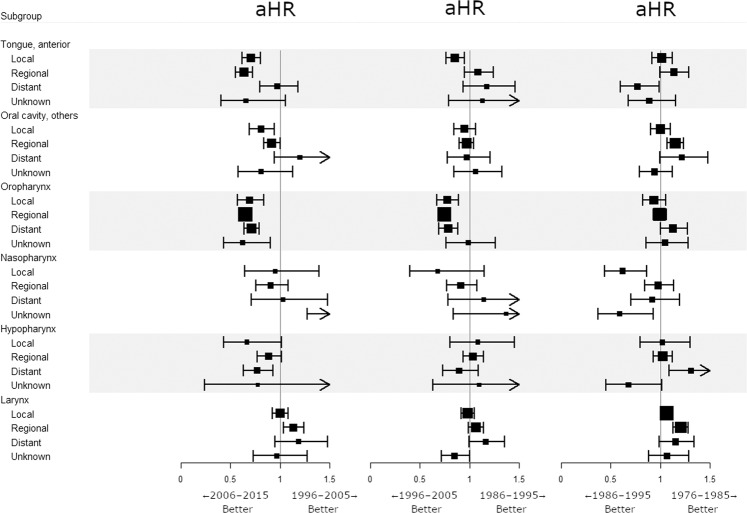
Effect of consecutive calendar decades on survival within disease extent variables stratified by primary sites. Multivariate adjustment was done to obtain each aHR as in Fig. 3. aHR, adjusted hazard ratio.

Compared with cancers of the other HNSCC sites, OTSCC and OPSCC shared similar trends in incidence and survival since the 2000s. However, unlike HPV-related OPSCC, the survival benefit in contemporary OTSCC might be attributed solely to clinical factors including, but not limited to, earlier diagnosis, refinement of treatment, and improvements in patient care and follow-up. Thus, we performed a multivariate analysis of RS adjusting for all variables (age, sex, race, tumor sites, grade, extent of disease, receipt of surgery, chemotherapy, and radiotherapy) for each subgroup. Adjusted hazard ratios (aHRs) for death were calculated for each primary site between two consecutive calendar decades. Multivariate analysis showed that since 2006, survival significantly improved for OTSCC (aHR = 0.697, 95% CI 0.642–0.757, *P* < 0.001) and OPSCC (aHR = 0.669, 95% CI 0.633–0.706, *P* < 0.001). Survival outcome for other-site HNSCCs has changed little during the 40-year period (Fig. 3). Further analysis showed that survival in OPSCC patients has gradually improved irrespective of surgery, chemotherapy, or radiotherapy since 1996 (Fig. 4). A similar treatment-independent survival increment has been observed only in OTSCC since 2006, but not in other oral cavity cancers. Except for OTSCC and OPSCC, since the 2000s, survival benefit if any was predominantly confined to patients who were treated using surgery, chemotherapy, or radiotherapy. Additional analysis was performed to stratify by disease extent. Since 1996, the survival for OPSCC has significantly increased regardless of disease extent (Fig. 5). Even the prognosis for patients diagnosed with metastatic OPSCC improved. During the same period, the survival benefit for OTSCC was confined to patients with only local or regional disease.

## Discussion

The current study showed that OTSCC shares similar incidence and survival features with OPSCC. The incidence of both cancers has been increasing, while that of HNSCC of other sites has been decreasing. Both OTSCC and OPSCC are characterized by excellent survival, and unlike that for other HNSCC, a trend of improved survival was observed for OTSCC and OPSCC, regardless of treatment or extent of disease.

Previous studies have reported the increasing incidence of oral tongue cancer^6,7^. Patel et al. demonstrated that oral tongue cancer might be an epidemiologically and biologically separate disease entity from other oral cavity cancers, similar to HPV-positive OPSCC being distinct from other HNSCC^7^.

So far, however, there has been no evidence that the rising trend of OTSCC is associated with HPV infection, as with OPSCC^19,20^. A recent meta-analysis reported that pooled HPV DNA prevalence estimates for oropharynx, oral cavity, and larynx (including hypopharynx) cancers were 45.8%, 24.2%, and 22.1%, respectively^21^. This study estimated the prevalence of HPV-related mobile tongue cancer as only 6.5%.

However, given that current HPV detection methods assess only the most common HPV genotypes, certain less common HPV types could be the potential causal factors of OTSCC^7,19^. Also, there may be a possibility that a completely unknown new etiology could underlie the recent increase in OTSCC. Further studies would be needed to be seen whether mechanisms underlying the increasing incidence of OTSCC exist and this increased OTSCC could be a new and separate disease entity.

To the best of our knowledge, this is the first report describing both the increasing incidence and the improving prognosis of OTSCC. Several studies demonstrated recent incidence and survival trends of head and neck cancer using the SEER database^2,3^. However, these studies did not focus on OTSCC, and the analysis period is limited. Our study analyzed the cancer incidence, RS, and CS so that more comprehensive evaluation is possible.

Our study found the incidence and survival features that are apparently shared by OTSCC and OPSCC. For both OTSCC and OPSCC, the timeframe of improved survival coincides with that of increasing incidence. Heightened awareness of the increasing incidence and excellent prognosis of OPSCC preceded the discovery of HPV-related OPSCC. Among HNSCC, OTSCC showed the best prognosis, which was second to only OPSCC. A 5-year CS greater than 90% suggests that OTSCC is, if properly treated, a curable condition. In contrast, HNSCC other than OTSCC and OPSCC had a 5-year CS < 90%. The enhanced prognosis of OTSCC might not be solely attributed to the advancement and refinement of treatment. We found that after adjusting for potential prognostic variables, survival outcome has improved for OTSCC and OPSCC since the 2000s regardless of treatment. In other HNSCC, survival enhancement was generally confined to patients receiving treatment. The favorable biology of HPV-related cancer leads to an excellent prognosis in OPSCC, irrespective of treatment. Thus, unadjusted, unknown variables like HPV in OPSCC could contribute to increased incidence and enhance prognosis in OTSCC in a treatment-independent fashion. Earlier detection of OTSCC might contribute to better prognosis. Early diagnosis enables optimal treatment to commence at less advanced cancer stages, potentially improving survival. The SEER data shows that the proportion of local disease extent has gradually increased in OTSCC over the four decades. We found that since the 2000s, survival in OTSCC was enhanced in patients with regional as well as local disease. The current study could not conclude whether early detection played a role in improving survival in OTSCC.

One study suggested that an increase in early-onset OTSCC may be associated with smokeless tobacco^22^. However, the main users of smokeless tobacco are men, and the use of smokeless tobacco among females has remained low from 1985 to 2015^23^, which is inconsistent with the fact that the increase in OTSCC is mainly observed in women^7,24^ and/or both the sexes^6^. Vered *et al*. insisted that the site-specific microenvironment may play a decisive role in poor prognosis of OTSCC, compared with that in base of the tongue cancer^25^. In the current study, the prognosis of OTSCC was almost comparable to that of OPSCC, suggesting that a novel, unknown etiology might drive the recent increase in OTSCC, whose pathogenesis and biology warrants classification as a distinct disease entity.

The current study is based on the analysis of incidence and outcome in HNSCC from the SEER dataset across four calendar decades. Several caveats should be noted while interpreting our observations. Most limitations of our study are related to the structure and shortcomings of the SEER dataset and have been reported previously^26^. Despite its comprehensiveness and statistically robust registry sizes, SEER lacks several key data items. As the SEER dataset does little to differentiate heterogeneous treatment cohorts, the recent advancements in surgery, chemotherapy, and radiotherapy are bound to be obscured by summary classification of treatments. Especially, the increasingly complex use of combined modality approaches is poorly captured by the current SEER program. The historical classification of disease extent is poignantly outdated compared with the current staging system, which not only addresses the anatomical disease burden in detail but also addresses the biological etiology^27^. Data on established risk factors, such as HPV status, Epstein-Barr virus infection, smoking, and alcohol consumption, are lacking in the SEER program. These limitations need to be considered while interpreting our findings from multivariate survival analysis.

In conclusion, the findings of this study support the previous observations regarding an increasing incidence of OTSCC and indicate that the trend coincides with significant survival improvement. Contrary to previous notion, the prognosis of contemporary OTSCC compares favorably with that of OPSCC. Since the 2000s, prognosis enhancement in both OTSCC and OPSCC is peculiar in that survival benefit is independent of treatment. On comparing HNSCC of various sites, we found that these incidence and survival characteristics are shared only by OTSCC and OPSCC. Contemporary OTSCC could be a harbinger of an emerging and distinct clinical entity, although future research is mandatory before clear conclusions can be drawn. Future investigations are needed to clarify the etiology of these observations. Though the observed variations in OTSCC may not be attributed to a novel causative agent, the increasing incidence and longer life expectancy provides further challenges for relevant health professionals. If the trend persists, an increasing number of OTSCC patients are expected to be recognized and treated accordingly. Given the findings of this study, vigilance needs to be exercised for early detection and diagnosis of OTSCC, and careful selection of optimal treatment is warranted to minimize long-term treatment toxicity.

## Methods

### Study population

All individuals diagnosed with HNSCC between 1976 and 2015 were identified from the SEER 9 database. All patients were subjected to pathological analysis and only those with histology of squamous cell neoplasms (International Classification of Diseases for Oncology, 3rd Edition [ICD-O-3] histology 8050–8089) were selected for this study. The included subsites according to the ICD-O-3/WHO 2008 were reclassified into five categories according to the Collaborative Stage schema v02.04 + (https://staging.seer.cancer.gov/cs/list/02.05.50/) as follows: (1) oral cavity (oral tongue, C020-C023, C028-C029; buccal mucosa, C060-C061; floor of mouth, C040-C041, C048-C049; gum, C030-C031, C039, C062; hard palate, C050; and others, C058-C059, C068-C069), (2) oropharynx (tonsil, C024, C090-C091, C098-C099, C14.2; base of tongue, C019; soft palate, C051, C052; oropharynx others, C100-C104, C108-C109), (3) nasopharynx (C110-C113, C118-C119), (4) hypopharynx (C129-C132, C138-C139), and (5) larynx (supraglottis, C321; glottis, C320; larynx others, C322–323, C328-C329). Lip cancer was excluded from the study because of an extremely favorable survival rate.

Patients were excluded if they were diagnosed based on their death certificate or autopsy or if there were no data on survival time. Patients with more than one primary tumor were also excluded (Supplementary Fig S1). The list of patients included for analysis was retrieved using the case listing option in the survival session of the SEER*Stat software (Ver 8.3.2) (https://seer.cancer.gov/seerstat/software/).

### Statistical analysis

APC and its statistical significance were analyzed using the Joinpoint Regression Program^28^. The Joinpoint Trend Analysis Software (Ver 4.7.0.0) can be downloaded from the following website: (https://surveillance.cancer.gov/joinpoint/download). From the SEER*Stat software, the age-adjusted incidence rate per 100,000 persons for each cancer was calculated. The results were then exported to the Joinpoint program to calculate APC. The program automatically calculated the optimal number of Joinpoint of the age-adjusted incidence rate graph for each tumor site and the statistical significance of APC. Any APC that was different from zero at the alpha level of 0.05 was considered statistically significant. RS was defined as the ratio of the proportion of observed survivors in a cohort of cancer patients to the proportion of expected survivors in a comparable set of individuals without cancer. RS adjusts for the race, sex, age, and the date at which the age was coded. The US Expected Survival Table 1970–2015 by individual year in the SEER*Stat software was used as the expected survival reference to create a rate table for use in the R software environment (Supplementary A). RS for various anatomical sites was calculated for up to 10 years after diagnosis. The Kaplan-Meier estimates of RS were calculated using the Pohar-Perme method^29^. A log-rank test was used for univariate analysis of Kaplan-Meier estimates. For multiple comparisons, Bonferroni correction was applied. Two-tailed *P* < 0.05/n was considered statistically significant, where *n* is the number of log-rank tests.

CS estimates were calculated RS and were directly obtained using the provided tool in the survival session of the SEER*Stat software. For multivariate analysis of RS, the multiplicative regression model for RS by Anderson et al. was used, which is an extension of the Cox proportional hazard function. The Anderson model reflexes changing trends in mortality, so that is particularly relevant in long-term follow-up studies^30^. This model was adjusted for the following variables: diagnostic periods, age, sex, race, cancer sites, extent of disease, grade, surgery, chemotherapy, and radiotherapy. Two-tailed *P* < 0.05 was considered statistically significant. RS analysis and graph generation were performed using R software version 3.5.1 (R Foundation for Statistical Computing, Vienna, Austria) and ‘relsurv’ R package^29^.

### Ethics approval and informed consent

As all information in the SEER database is de-identified, the current study was exempt from institutional review board approval, and informed consent was not required. The study was performed in accordance with the Declaration of Helsinki.

## Supplementary information

supplementary tables.

supplementary figures.

Supplementary A.

## Data Availability

The SEER data used in our study is available at https://seer.cancer.gov/seertrack/data/request/.

## References

[1] Siegel RL, Miller KD, Jemal A (2019). Cancer statistics, 2019. CA: a cancer journal for clinicians.

[2] Mourad M (2017). Epidemiological trends of head and neck cancer in the United States: a SEER population study. Journal of Oral and Maxillofacial Surgery.

[3] Mahal BA (2019). Incidence and Demographic Burden of HPV-Associated Oropharyngeal Head and Neck Cancers in the United States. Cancer Epidemiology and Prevention. Biomarkers.

[4] Brennan JA (1995). Association between cigarette smoking and mutation of the p53 gene in squamous-cell carcinoma of the head and neck. New England Journal of Medicine.

[5] Sturgis EM, Cinciripini PM (2007). Trends in head and neck cancer incidence in relation to smoking prevalence: an emerging epidemic of human papillomavirus‐associated cancers? Cancer: Interdisciplinary International. Journal of the American Cancer Society.

[6] Tota JE (2017). Rising incidence of oral tongue cancer among white men and women in the United States, 1973–2012. Oral oncology.

[7] Patel SC (2011). Increasing incidence of oral tongue squamous cell carcinoma in young white women, age 18 to 44 years. Journal of Clinical Oncology.

[8] Ng JH, Iyer NG, Tan MH, Edgren G (2017). Changing epidemiology of oral squamous cell carcinoma of the tongue: A global study. Head & neck.

[9] Westra WH (2009). The changing face of head and neck cancer in the 21st century: the impact of HPV on the epidemiology and pathology of oral cancer. Head and neck pathology.

[10] Marur S, D’Souza G, Westra WH, Forastiere AA (2010). HPV-associated head and neck cancer: a virus-related cancer epidemic. The lancet oncology.

[11] Smith EM (2004). Age, sexual behavior and human papillomavirus infection in oral cavity and oropharyngeal cancers. International journal of cancer.

[12] Chaturvedi AK, Engels EA, Anderson WF, Gillison ML (2008). Incidence trends for human papillomavirus-related and-unrelated oral squamous cell carcinomas in the United States. Journal of clinical oncology.

[13] Kimple RJ (2013). Enhanced radiation sensitivity in HPV-positive head and neck cancer. Cancer research.

[14] Lv J-W (2018). A national study of survival trends and conditional survival in nasopharyngeal carcinoma: analysis of the national population-based surveillance epidemiology and end results registry. Cancer research and treatment: official journal of Korean Cancer Association.

[15] Petersen JF (2018). Trends in treatment, incidence and survival of hypopharynx cancer: a 20-year population-based study in the Netherlands. European Archives of Oto-Rhino-Laryngology.

[16] Nahavandipour A (2019). Incidence and survival of laryngeal cancer in Denmark: a nation-wide study from 1980 to 2014. Acta Oncologica.

[17] Bates JE (2019). Locally advanced hypopharyngeal and laryngeal cancer: Influence of HPV status. Radiotherapy and Oncology.

[18] Burr, A. R. et al. HPV impacts survival of stage IVC non-oropharyngeal HNSCC cancer patients. *Otorhinolaryngology-head and neck surgery* **3** (2018). 10.15761/OHNS.1000160 PMC6157736 30271885

[19] Liang X-H, Lewis J, Foote R, Smith D, Kademani D (2008). Prevalence and significance of human papillomavirus in oral tongue cancer: the Mayo Clinic experience. Journal of Oral and Maxillofacial Surgery.

[20] Salem, A. Dismissing links between HPV and aggressive tongue cancer in young patients. *Annals of oncology* **21** (2010). 10.1093/annonc/mdp380 19825879

[21] Ndiaye C (2014). HPV DNA, E6/E7 mRNA, and p16INK4a detection in head and neck cancers: a systematic review and meta-analysis. The Lancet Oncology.

[22] Campbell BR (2018). Early onset oral tongue cancer in the United States: A literature review. Oral oncology.

[23] U.S. Department of Health and Human Services. The Health Consequences of Smoking—50 Years of Progress: A Report of the Surgeon General. Atlanta: U.S. Department of Health and Human Services, Centers for Disease Control and Prevention, National Center for Chronic Disease Prevention and Health Promotion, Office on Smoking and Health, (2014).

[24] Joseph LJ (2015). Racial disparities in squamous cell carcinoma of the oral tongue among women: a SEER data analysis. Oral Oncology.

[25] Vered M, Dayan D, Salo T (2011). The role of the tumour microenvironment in the biology of head and neck cancer: lessons from mobile tongue cancer. Nature Reviews Cancer.

[26] Fuller CD (2007). Conditional survival in head and neck squamous cell carcinoma: results from the SEER dataset 1973–1998. Cancer: Interdisciplinary International. Journal of the American Cancer Society.

[27] Lydiatt WM (2017). Head and neck cancers—major changes in the American Joint Committee on cancer eighth edition cancer staging manual. CA: a cancer journal for clinicians.

[28] Kim HJ, Fay MP, Feuer EJ, Midthune DN (2000). Permutation tests for joinpoint regression with applications to cancer rates. Statistics in medicine.

[29] Perme MP, Pavlic K (2018). Nonparametric Relative Survival Analysis with the R Package relsurv. Journal of Statistical Software.

[30] Andersen PK (1985). A Cox regression model for the relative mortality and its application to diabetes mellitus survival data. Biometrics.

