# Supplementary material for: Increasing incidence and improving survival of oral tongue squamous cell carcinoma
Source: Sci Rep. 2020 May 12;10:7877. doi: 10.1038/s41598-020-64748-0 (PMC7217912; doi:10.1038/s41598-020-64748-0)
Supplement: Supplementary file 1 — supplementary tables. [file 41598_2020_64748_MOESM1_ESM.docx]

**Supplementary material**

**Increasing incidence and improving survival of oral tongue squamous cell carcinoma**

Yi-Jun Kim MD^1,2,3^, Jin Ho Kim MD, PhD^2*^

^1^Center for Precision Medicine, Seoul National University Hospital, Seoul, Republic of Korea

^2^Department of Radiation Oncology, Seoul National University College of Medicine, Seoul, Republic of Korea

^3^Graduate School of Medicine, College of Medicine, Ewha Womans University, Seoul, Republic of Korea

Table S1. Relative survival of OTSCC in the SEER database according to the clinical features and diagnostic period

| Characteristics | 1976–1985 | | 1986–1995 | | 1996–2005 | | 2006–2015 | |
| --- | --- | --- | --- | --- | --- | --- | --- | --- |
|  | n=1711 | | n=1840 | | n=2132 | | n=3277 | |
|  | 5-yr RS (%) | (95% CI) | 5-yr RS (%) | (95% CI) | 5-yr RS (%) | (95% CI) | 5-yr RS (%) | (95% CI) |
| Extent of disease |  |  |  |  |  |  |  |  |
| Local | 50.8 | (47.6–54.3) | 57.7 | (54.7–60.9) | 65.1 | (62.4–67.9) | 78.2 | (76.1–80.4) |
| Regional | 22.8 | (19.6–26.5) | 23.8 | (20.4–27.7) | 29.5 | (26.1–33.3) | 45.1 | (41.7–48.7) |
| Distant | 11.5 | (7.2–18.5) | 17.9 | (13.1–24.4) | 16.6 | (12.5–22.0) | 23.9 | (19.5–29.2) |
| Unknown/blank(s) | 28.1 | (21.4–36.8) | 39.8 | (32.5–48.6) | 40.1 | (30.8–52.3) | 58.2 | (47.7–70.8) |
| Grade |  |  |  |  |  |  |  |  |
| Well differentiated | 42.6 | (38.5–47.2) | 51.8 | (47.4–56.6) | 62.8 | (58.6–67.3) | 75.0 | (71.5–78.6) |
| Moderately differentiated | 37.3 | (33.6–41.4) | 39.0 | (35.6–42.6) | 45.4 | (42.5–48.6) | 59.2 | (56.6–62.0) |
| Poorly differentiated | 22.0 | (16.9–28.7) | 31.1 | (26.2–37.1) | 35.2 | (30.5–40.5) | 47.2 | (42.6–52.2) |
| Undifferentiated, anaplastic | 16.7 | (4.6–61.6) | NA | NA | 50.1 | (24.7–100.0) | 60.0 | (32.1–100.0) |
| Unknown | 36.8 | (32.4–41.8) | 49.0 | (43.9–54.7) | 52.8 | (46.9–59.4) | 64.6 | (59.7–70.0) |
| Surgery |  |  |  |  |  |  |  |  |
| No/unknown | 17.2 | (14.2–20.8) | 18.5 | (15.3–22.6) | 17.1 | (13.7–21.4) | 29.6 | (25.6–34.3) |
| Yes | 45.1 | (42.3–48.0) | 50.0 | (47.4–52.7) | 55.3 | (53.0–57.7) | 67.6 | (65.6–69.6) |
| Chemotherapy |  |  |  |  |  |  |  |  |
| No/unknown | 40.1 | (37.7–42.7) | 46.2 | (43.9–48.7) | 52.4 | (50.2–54.8) | 68.3 | (66.3–70.3) |
| Yes | 9.3 | (6.0–14.6) | 13.4 | (9.4–19.0) | 22.3 | (17.9–27.8) | 39.5 | (35.8–43.5) |
| Radiotherapy |  |  |  |  |  |  |  |  |
| No/unknown | 47.4 | (44.2–50.8) | 55.8 | (52.8–58.9) | 61.2 | (58.5–64.1) | 71.0 | (68.8–73.3) |
| Yes | 24.9 | (22.1–28.1) | 25.9 | (23.0–29.1) | 31.8 | (28.9–35.0) | 46.5 | (43.6–49.7) |

Abbreviations: OTSCC, oral tongue squamous cell carcinoma; SEER, Surveillance, Epidemiology, and End Results; RS, relative survival; CI, confidence interval.

Table S2. Relative survival of non-oral tongue oral cavity SCC in the SEER database according to the clinical features and diagnostic period

| Characteristics | 1976–1985 | | 1986–1995 | | 1996–2005 | | 2006–2015 | |
| --- | --- | --- | --- | --- | --- | --- | --- | --- |
|  | n=3340 | | n=2906 | | n=2571 | | n=2737 | |
|  | 5-yr RS (%) | (95% CI) | 5-yr RS (%) | (95% CI) | 5-yr RS (%) | (95% CI) | 5-yr RS (%) | (95% CI) |
| Extent of disease |  |  |  |  |  |  |  |  |
| Local | 55.5 | (52.5–58.7) | 57.1 | (53.8–60.6) | 59.2 | (55.8–62.8) | 68.8 | (65.3–72.4) |
| Regional | 30.7 | (28.6–32.9) | 28.3 | (26.2–30.7) | 30.7 | (28.4–33.1) | 38.4 | (35.7–41.2) |
| Distant | 13.4 | (10.0–18.0) | 13.4 | (9.6–18.7) | 15.0 | (10.5–21.4) | 14.3 | (10.1–20.4) |
| Unknown/blank(s) | 25.9 | (21.4–31.4) | 30.2 | (25.2–36.1) | 32.0 | (25.2–40.8) | 36.6 | (27.6–48.4) |
| Grade |  |  |  |  |  |  |  |  |
| Well differentiated | 42.2 | (39.0–45.5) | 43.1 | (39.6–46.9) | 48.4 | (44.2–53.1) | 56.5 | (52.4–61.0) |
| Moderately differentiated | 35.6 | (32.9–38.5) | 34.2 | (31.6–37.0) | 38.1 | (35.5–40.9) | 44.4 | (41.5–47.5) |
| Poorly differentiated | 21.0 | (17.0–25.9) | 26.0 | (22.2–30.6) | 26.5 | (22.7–30.9) | 34.1 | (29.4–39.7) |
| Undifferentiated, anaplastic | 32.3 | (19.3–54.2) | 14.4 | (3.6–58.0) | 16.7 | (5.7–49.2) | 53.5 | (29.9–95.8) |
| Unknown | 37.2 | (34.2–40.5) | 37.0 | (33.3–41.2) | 39.4 | (34.7–44.7) | 46.5 | (41.1–52.7) |
| Surgery |  |  |  |  |  |  |  |  |
| No/unknown | 19.7 | (17.4–22.2) | 15.8 | (13.5–18.6) | 15.3 | (12.7–18.4) | 16.1 | (13.3–19.6) |
| Yes | 44.1 | (42.1–46.2) | 42.8 | (40.8–45.0) | 45.5 | (43.3–47.8) | 54.8 | (52.4–57.3) |
| Chemotherapy |  |  |  |  |  |  |  |  |
| No/unknown | 38.7 | (37.0–40.5) | 38.8 | (37.0–40.8) | 40.8 | (38.8–42.9) | 51.2 | (48.8–53.7) |
| Yes | 13.4 | (10.2–17.7) | 11.3 | (8.4–15.2) | 20.5 | (16.7–25.3) | 30.2 | (26.6–34.3) |
| Radiotherapy |  |  |  |  |  |  |  |  |
| No/unknown | 47.1 | (44.8–49.6) | 47.3 | (44.8–50.0) | 48.2 | (45.5–51.1) | 55.3 | (52.5–58.2) |
| Yes | 25.3 | (23.3–27.5) | 24.4 | (22.3–26.7) | 28.3 | (26.0–30.9) | 35.1 | (32.2–38.2) |

Abbreviations: SCC, squamous cell carcinoma; RS, relative survival; CI, confidence interval.

Table S3. Relative survival of OPSCC in the SEER database according to the clinical features and diagnostic period

| Characteristics | 1976–1985 | | 1986–1995 | | 1996–2005 | | 2006–2015 | |
| --- | --- | --- | --- | --- | --- | --- | --- | --- |
|  | n=3661 | | n=3753 | | n=5176 | | n=9266 | |
|  | 5-yr RS (%) | (95% CI) | 5-yr RS (%) | (95% CI) | 5-yr RS (%) | (95% CI) | 5-yr RS (%) | (95% CI) |
| Extent of disease |  |  |  |  |  |  |  |  |
| Local | 39.0 | (35.5–42.9) | 44.5 | (40.6–48.8) | 59.5 | (55.5–63.7) | 74.6 | (71.1–78.2) |
| Regional | 24.4 | (22.6–26.4) | 32.1 | (30.2–34.0) | 53.1 | (51.5–54.8) | 71.4 | (70.2–72.7) |
| Distant | 11.4 | (9.3–13.9) | 13.3 | (10.7–16.5) | 27.0 | (24.0–30.3) | 46.1 | (43.7–48.7) |
| Unknown/blank(s) | 25.9 | (20.8–32.1) | 25.5 | (20.3–32.0) | 28.0 | (21.237.0) | 51.8 | (43.0–62.4) |
| Grade |  |  |  |  |  |  |  |  |
| Well differentiated | 28.2 | (24.5–32.5) | 26.9 | (22.7–32.0) | 49.9 | (44.0–56.6) | 56.9 | (51.3–63.2) |
| Moderately differentiated | 22.6 | (20.5–25.0) | 30.1 | (27.8–32.5) | 45.9 | (43.7–48.2) | 62.8 | (60.9–64.9) |
| Poorly differentiated | 25.7 | (22.9–28.7) | 34.9 | (32.3–37.7) | 55.3 | (53.1–57.6) | 71.8 | (70.2–73.5) |
| Undifferentiated, anaplastic | 28.6 | (18.6–43.9) | 43.0 | (31.4–58.9) | 65.3 | (54.4–78.4) | 75.1 | (65.2–86.4) |
| Unknown | 24.6 | (21.9–27.5) | 26.1 | (22.9–29.8) | 43.0 | (40.0–46.2) | 62.7 | (60.6–64.9) |
| Surgery |  |  |  |  |  |  |  |  |
| No/unknown | 17.9 | (16.4–19.6) | 21.8 | (20.1–23.7) | 37.8 | (36.0–39.7) | 59.5 | (58.2–61.0) |
| Yes | 34.5 | (32.1–37.0) | 40.8 | (38.6–43.2) | 61.4 | (59.5–63.3) | 78.1 | (76.5–79.7) |
| Chemotherapy |  |  |  |  |  |  |  |  |
| No/unknown | 27.1 | (25.6–28.8) | 34.1 | (32.4–35.9) | 46.3 | (44.4–48.2) | 61.0 | (58.9–63.2) |
| Yes | 13.4 | (11.0–16.2) | 21.2 | (18.8–24.0) | 52.9 | (50.9–54.9) | 68.1 | (66.8–69.3) |
| Radiotherapy |  |  |  |  |  |  |  |  |
| No/unknown | 30.3 | (27.3–33.6) | 27.5 | (24.6–30.8) | 37.5 | (34.4–40.8) | 44.0 | (41.2–47.1) |
| Yes | 23.0 | (21.5–24.6) | 31.8 | (30.1–33.5) | 51.9 | (50.4–53.4) | 69.9 | (68.8–71.1) |

Abbreviations: OPSCC, oropharyngeal squamous cell carcinoma; RS, relative survival; CI, confidence interval.

Table S4. Relative survival of nasopharyngeal SCC in the SEER database according to the clinical features and diagnostic period

| Characteristics | 1976–1985 | | 1986–1995 | | 1996–2005 | | 2006–2015 | |
| --- | --- | --- | --- | --- | --- | --- | --- | --- |
|  | n=806 | | n=843 | | n=873 | | n=924 | |
|  | 5-yr RS (%) | (95% CI) | 5-yr RS (%) | (95% CI) | 5-yr RS (%) | (95% CI) | 5-yr RS (%) | (95% CI) |
| Extent of disease |  |  |  |  |  |  |  |  |
| Local | 47.3 | (39.7–56.2) | 64.1 | (55.3–74.3) | 56.3 | (47.8–66.2) | 63.8 | (56.3–72.2) |
| Regional | 37.6 | (33.2–42.7) | 43.4 | (39.4–47.8) | 54.3 | (50.6–58.3) | 60.9 | (56.7–65.4) |
| Distant | 23.0 | (17.7–29.9) | 22.4 | (16.3–30.6) | 28.3 | (20.3–39.3) | 20.3 | (13.3–31.2) |
| Unknown/blank(s) | 27.9 | (19.2–40.5) | 38.8 | (28.2–53.3) | 43.7 | (29.1–65.7) | 49.8 | (35.8–69.1) |
| Grade |  |  |  |  |  |  |  |  |
| Well differentiated | 12.9 | (5.8–28.8) | 27.0 | (14.9–48.8) | 40.1 | (22.5–71.3) | 56.4 | (37.4–85.0) |
| Moderately differentiated | 25.8 | (18.7–35.7) | 24.5 | (17.4–34.7) | 35.6 | (27.2–46.5) | 40.5 | (31.9–51.6) |
| Poorly differentiated | 39.0 | (34.4–44.3) | 42.8 | (38.4–47.6) | 49.2 | (44.4–54.4) | 58.0 | (52.3–64.4) |
| Undifferentiated, anaplastic | 24.0 | (14.6–39.3) | 59.2 | (49.3–71.0) | 64.3 | (57.2–72.3) | 69.3 | (61.8–77.6) |
| Unknown | 37.9 | (32.3–44.5) | 44.8 | (38.2–52.7) | 54.8 | (48.4–61.9) | 54.0 | (48.2–60.5) |
| Surgery |  |  |  |  |  |  |  |  |
| No/unknown | 33.3 | (29.9–37.1) | 41.9 | (38.4–45.7) | 50.5 | (46.9–54.5) | 55.9 | (52.2–59.7) |
| Yes | 44.0 | (36.3–53.3) | 43.9 | (36.0–53.5) | 55.4 | (48.8–63.0) | 64.5 | (54.6–76.1) |
| Chemotherapy |  |  |  |  |  |  |  |  |
| No/unknown | 36.5 | (33.1–40.4) | 42.1 | (38.1–46.5) | 42.0 | (35.7–49.4) | 37.6 | (30.0–47.2) |
| Yes | 27.8 | (21.1–36.5) | 42.3 | (37.0–48.5) | 54.6 | (50.9–58.5) | 60.3 | (56.6–64.2) |
| Radiotherapy |  |  |  |  |  |  |  |  |
| No/unknown | 24.9 | (17.8–34.9) | 21.1 | (13.9–32.0) | 32.1 | (23.1–44.6) | 17.2 | (10.8–27.4) |
| Yes | 36.5 | (33.1–40.3) | 44.5 | (41.0–48.2) | 53.5 | (50.1–57.1) | 62.2 | (58.6–66.1) |

Abbreviations: SCC, squamous cell carcinoma; RS, relative survival; CI, confidence interval.

Table S5. Relative survival of hypopharyngeal SCC in the SEER database according to the clinical features and diagnostic period

| Characteristics | 1976–1985 | | 1986–1995 | | 1996–2005 | | 2006–2015 | |
| --- | --- | --- | --- | --- | --- | --- | --- | --- |
|  | n=1599 | | n=1530 | | n=1201 | | n=1031 | |
|  | 5-yr RS (%) | (95% CI) | 5-yr RS (%) | (95% CI) | 5-yr RS (%) | (95% CI) | 5-yr RS (%) | (95% CI) |
| Extent of disease |  |  |  |  |  |  |  |  |
| Local | 25.5 | (20.4–31.8) | 38.5 | (31.4–47.2) | 27.6 | (20.0–38.0) | 54.2 | (43.0–68.3) |
| Regional | 15.3 | (13.1–17.8) | 18.8 | (16.6–21.3) | 20.8 | (18.2–23.8) | 31.2 | (27.1–36.0) |
| Distant | 9.0 | (6.5–12.4) | 5.6 | (3.5–9.2) | 12.2 | (8.8–16.8) | 26.8 | (22.4–32.1) |
| Unknown/blank(s) | 6.8 | (3.1–14.7) | 22.5 | (14.2–35.7) | 17.9 | (8.5–37.6) | 25.2 | (8.4–75.2) |
| Grade |  |  |  |  |  |  |  |  |
| Well differentiated | 14.7 | (10.2–21.3) | 14.1 | (8.8–22.5) | 20.9 | (12.3–35.6) | 34.9 | (22.3–54.6) |
| Moderately differentiated | 16.6 | (13.8–20.0) | 19.8 | (17.0–23.1) | 17.6 | (14.5–21.3) | 30.6 | (25.8–36.2) |
| Poorly differentiated | 13.0 | (10.3–16.4) | 20.8 | (17.6–24.5) | 22.6 | (19.0–26.8) | 32.0 | (26.9–38.1) |
| Undifferentiated, anaplastic | 9.1 | (2.9–28.5) | 8.4 | (2.1–33.9) | 17.9 | (7.1–45.4) | 15.7 | (4.0–61.8) |
| Unknown | 15.2 | (12.0–19.4) | 12.4 | (8.7–17.7) | 16.7 | (12.2–22.8) | 32.5 | (26.7–39.6) |
| Surgery |  |  |  |  |  |  |  |  |
| No/unknown | 9.5 | (7.7–11.8) | 12.7 | (10.6–15.3) | 16.3 | (13.8–19.2) | 29.2 | (26.0–32.7) |
| Yes | 20.0 | (17.4–23.0) | 24.8 | (21.9–28.1) | 24.4 | (20.7–28.7) | 41.8 | (34.0–51.3) |
| Chemotherapy |  |  |  |  |  |  |  |  |
| No/unknown | 17.0 | (15.1–19.2) | 21.4 | (19.1–24.0) | 16.1 | (13.5–19.3) | 23.1 | (17.8–30.1) |
| Yes | 5.2 | (3.2–8.4) | 11.9 | (9.3–15.3) | 23.0 | (19.8–26.7) | 34.1 | (30.5–38.1) |
| Radiotherapy |  |  |  |  |  |  |  |  |
| No/unknown | 16.0 | (12.6–20.2) | 12.5 | (9.3–16.8) | 8.0 | (5.2–12.3) | 16.9 | (11.6–24.4) |
| Yes | 14.6 | (12.7–16.7) | 20.2 | (18.0–22.6) | 22.2 | (19.7–25.0) | 34.5 | (31.1–38.3) |

Abbreviations: SCC, squamous cell carcinoma; RS, relative survival; CI, confidence interval.

Table S6. Relative survival of laryngeal SCC in the SEER database according to the clinical features and diagnostic period

| Characteristics | 1976–1985 | | 1986–1995 | | 1996–2005 | | 2006–2015 | |
| --- | --- | --- | --- | --- | --- | --- | --- | --- |
|  | n=6181 | | n=5949 | | n=5390 | | n=5983 | |
|  | 5-yr RS (%) | (95% CI) | 5-yr RS (%) | (95% CI) | 5-yr RS (%) | (95% CI) | 5-yr RS (%) | (95% CI) |
| Extent of disease |  |  |  |  |  |  |  |  |
| Local | 65.4 | (63.8–67.1) | 65.1 | (63.4–66.8) | 66.1 | (64.3–67.9) | 66.8 | (65.1–68.5) |
| Regional | 38.6 | (36.6–40.7) | 35.8 | (33.8–37.8) | 36.8 | (34.7–38.9) | 35.8 | (33.2–38.5) |
| Distant | 19.9 | (16.7–23.7) | 19.7 | (16.3–23.9) | 19.2 | (15.6–23.7) | 8.6 | (5.3–13.9) |
| Unknown/blank(s) | 44.0 | (38.5–50.4) | 44.9 | (39.3–51.3) | 51.2 | (44.1–59.5) | 55.3 | (48.8–62.6) |
| Grade |  |  |  |  |  |  |  |  |
| Well differentiated | 62.2 | (59.6–64.8) | 60.1 | (57.3–63.1) | 70.4 | (67.2–73.8) | 71.4 | (67.9–75.0) |
| Moderately differentiated | 49.7 | (47.6–51.8) | 50.3 | (48.4–52.3) | 48.9 | (46.9–50.9) | 56.1 | (54.1–58.2) |
| Poorly differentiated | 35.3 | (32.5–38.3) | 35.1 | (32.3–38.0) | 37.1 | (34.3–40.1) | 42.7 | (39.5–46.2) |
| Undifferentiated, anaplastic | 38.0 | (26.4–54.7) | 29.9 | (19.5–45.9) | 25.2 | (14.2–44.7) | 32.4 | (16.1–65.5) |
| Unknown | 54.1 | (51.6–56.7) | 54.8 | (51.9–57.8) | 56.6 | (53.6–59.8) | 58.7 | (55.8–61.7) |
| Surgery |  |  |  |  |  |  |  |  |
| No/unknown | 49.2 | (47.3–51.2) | 46.5 | (44.6–48.5) | 47.5 | (45.7–49.4) | 52.0 | (50.3–53.9) |
| Yes | 52.5 | (50.9–54.2) | 52.5 | (50.8–54.2) | 54.7 | (52.8–56.7) | 63.3 | (61.1–65.5) |
| Chemotherapy |  |  |  |  |  |  |  |  |
| No/unknown | 52.9 | (51.6–54.2) | 52.6 | (51.3–54) | 56.0 | (54.4–57.5) | 63.3 | (61.6–65.1) |
| Yes | 17.1 | (13.4–21.8) | 24.7 | (21.5–28.5) | 35.0 | (32.5–37.7) | 44.1 | (41.8–46.5) |
| Radiotherapy |  |  |  |  |  |  |  |  |
| No/unknown | 54.0 | (51.9–56.3) | 47.6 | (45.0–50.2) | 48.1 | (45.2–51.2) | 50.4 | (47.4–53.5) |
| Yes | 49.7 | (48.2–51.2) | 50.7 | (49.3–52.2) | 51.7 | (50.2–53.2) | 57.9 | (56.4–59.6) |

Abbreviations: SCC, squamous cell carcinoma; RS, relative survival; CI, confidence interval.
