# Supplementary material for: Increasing incidence and improving survival of oral tongue squamous cell carcinoma
Source: Sci Rep. 2020 May 12;10:7877. doi: 10.1038/s41598-020-64748-0 (PMC7217912; doi:10.1038/s41598-020-64748-0)

**Supplementary material**

**Increasing incidence and improving survival of oral tongue squamous cell carcinoma**

Yi-Jun Kim MD^1,2,3^, Jin Ho Kim MD, PhD^2*^

^1^Center for Precision Medicine, Seoul National University Hospital, Seoul, Republic of Korea

^2^Department of Radiation Oncology, Seoul National University College of Medicine, Seoul, Republic of Korea

^3^Graduate School of Medicine, College of Medicine, Ewha Womans University, Seoul, Republic of Korea

Figure S1. Flow chart of patients included in the study


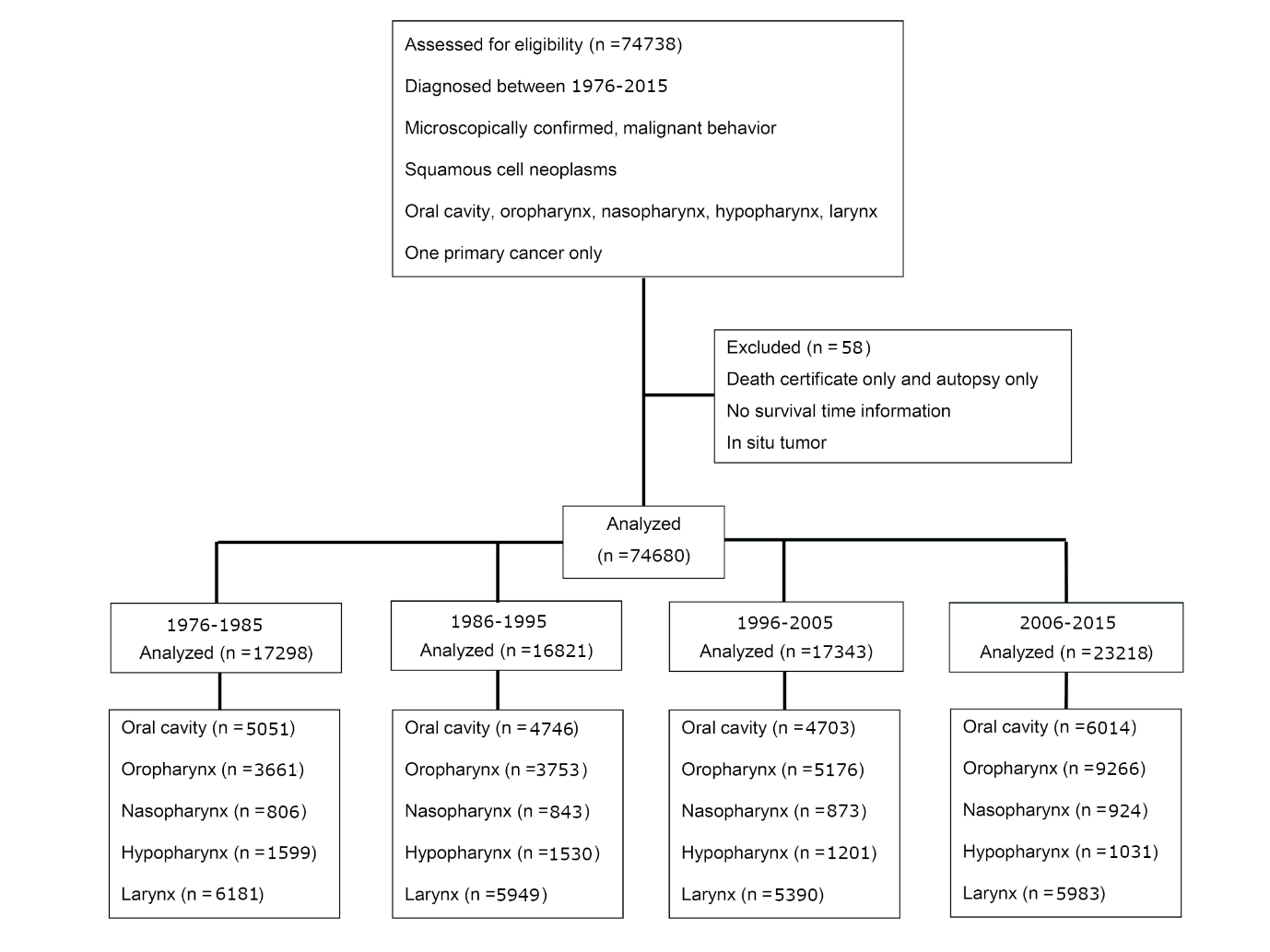


Figure S2. Relative survival (RS) and 5-year conditional survival (CS) estimates for head and neck squamous cell carcinoma according to each anatomical site (nasopharynx, hypopharynx, and larynx) during the four calendar periods. For each calendar decade, a series of CSs was provided at the consecutive post-diagnosis years. Asterisks represent statistical significance after adjustment by the Bonferroni correction (P<0.05/6).


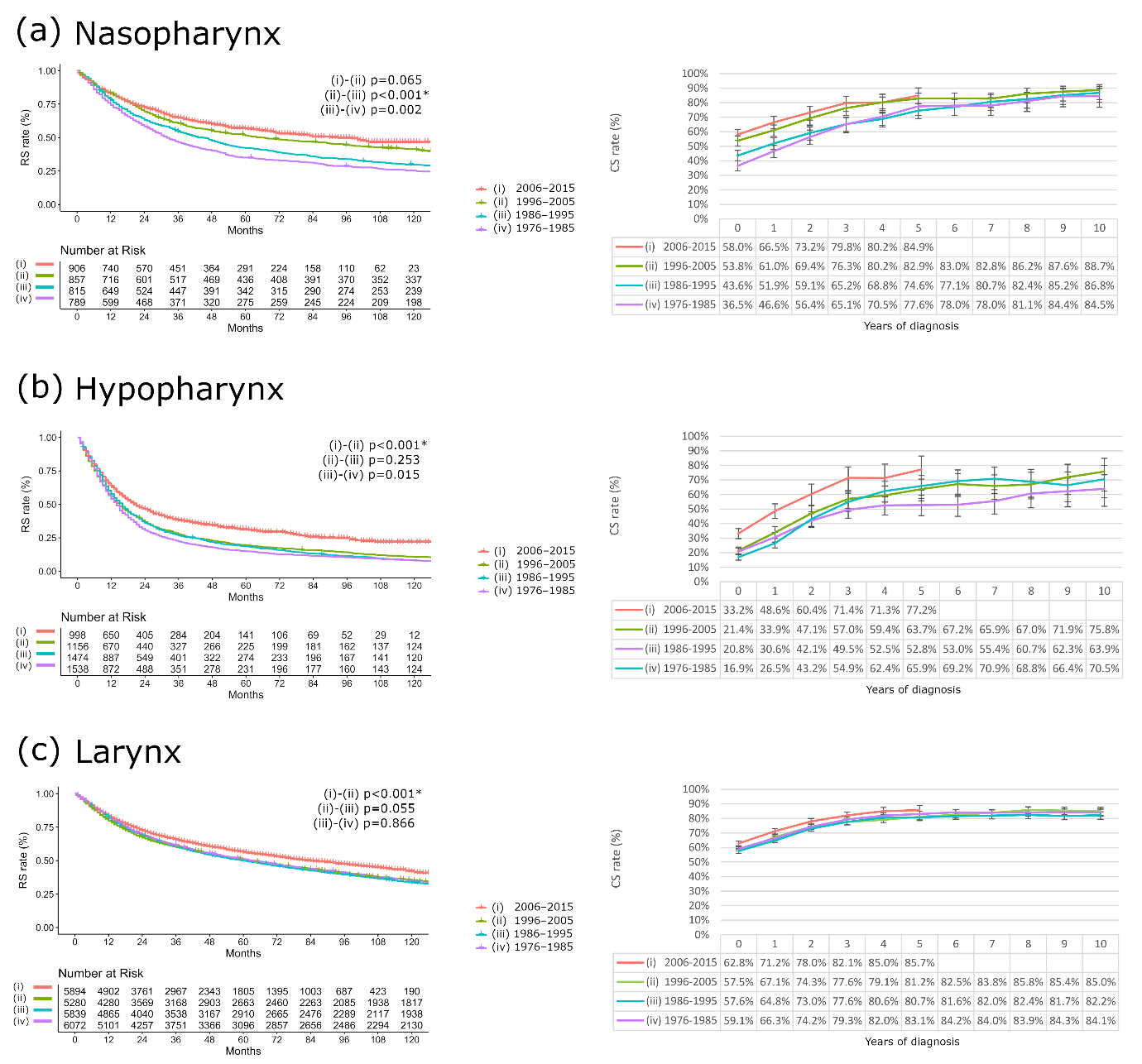

Supplement: Supplementary file 2 — supplementary figures. [file 41598_2020_64748_MOESM2_ESM.docx]
