# Supplementary material for: Increasing incidence and improving survival of oral tongue squamous cell carcinoma
Source: Sci Rep. 2020 May 12;10:7877. doi: 10.1038/s41598-020-64748-0 (PMC7217912; doi:10.1038/s41598-020-64748-0)
Supplement: Supplementary file 3 — Supplementary A. [file 41598_2020_64748_MOESM3_ESM.docx]

**Supplementary material**

**Increasing incidence and improving survival of oral tongue squamous cell carcinoma**

Yi-Jun Kim MD^1,2,3^, Jin Ho Kim MD, PhD^2*^

^1^Center for Precision Medicine, Seoul National University Hospital, Seoul, Republic of Korea

^2^Department of Radiation Oncology, Seoul National University College of Medicine, Seoul, Republic of Korea

^3^Graduate School of Medicine, College of Medicine, Ewha Womans University, Seoul, Republic of Korea

| lifetable <- read.delim2(file="https://seer.cancer.gov/expsurvival/US.1970thru2015.individual.years.txt", header = FALSE)  head(lifetable) |
| --- |
|  |
| # install.packages("tidyr") |
| library(tidyr) |
| test <- tidyr::extract(lifetable, V1, c("age", "sex", "race", "year", "survival"), |
| regex="([0-9]{3})([0-9]{1})([0-9]{2})([0-9]{4})............([0-9]{7})") |
| head(test) |
|  |
| test$age <- as.integer(test$age) |
| test$sex <- as.integer(test$sex) |
| test$race <- as.integer(test$race) |
| test$year <- as.integer(test$year) |
| test$survival <- as.integer(test$survival) |
| head(test) |
| test$survival <- test$survival/1000000 |
| head(test) |
| lifetable <- test |
|  |
| test2 <- tidyr::spread(lifetable, year, survival) |
| head(test2) |
| lifetable <- test2 |
|  |
| # reference... |
| # https://stackoverflow.com/questions/31064599/r-making-the-rate-table-for-relative-survival-analysis |
|  |
| lt_ss_f <- subset(lifetable, race ==1 & sex ==2); lt_ss_f <- lt_ss_f[-c(1:3)]; lt_ss_f <- as.matrix(lt_ss_f) |
| lt_ss_m <- subset(lifetable, race ==1 & sex ==1); lt_ss_m <- lt_ss_m[-c(1:3)]; lt_ss_m <- as.matrix(lt_ss_m) |
| assign(paste("race", 1, sep=""), transrate(lt_ss_m, lt_ss_f, yearlim=c(1970,2015), int.length=1)) |
|  |
| lt_ss_f <- subset(lifetable, race ==2 & sex ==2); lt_ss_f <- lt_ss_f[-c(1:3)]; lt_ss_f <- as.matrix(lt_ss_f) |
| lt_ss_m <- subset(lifetable, race ==2 & sex ==1); lt_ss_m <- lt_ss_m[-c(1:3)]; lt_ss_m <- as.matrix(lt_ss_m) |
| assign(paste("race", 2, sep=""), transrate(lt_ss_m, lt_ss_f, yearlim=c(1970,2015), int.length=1)) |
|  |
| lt_ss_f <- subset(lifetable, race ==3 & sex ==2); lt_ss_f <- lt_ss_f[-c(1:3)]; lt_ss_f <- as.matrix(lt_ss_f) |
| lt_ss_m <- subset(lifetable, race ==3 & sex ==1); lt_ss_m <- lt_ss_m[-c(1:3)]; lt_ss_m <- as.matrix(lt_ss_m) |
| assign(paste("race", 3, sep=""), transrate(lt_ss_m, lt_ss_f, yearlim=c(1970,2015), int.length=1)) |
|  |
| lt_ss_f <- subset(lifetable, race ==7 & sex ==2); lt_ss_f <- lt_ss_f[-c(1:3)]; lt_ss_f <- as.matrix(lt_ss_f) |
| lt_ss_m <- subset(lifetable, race ==7 & sex ==1); lt_ss_m <- lt_ss_m[-c(1:3)]; lt_ss_m <- as.matrix(lt_ss_m) |
| assign(paste("race", 7, sep=""), transrate(lt_ss_m, lt_ss_f, yearlim=c(1970,2015), int.length=1)) |
|  |
| lt_ss_f <- subset(lifetable, race ==9 & sex ==2); lt_ss_f <- lt_ss_f[-c(1:3)]; lt_ss_f <- as.matrix(lt_ss_f) |
| lt_ss_m <- subset(lifetable, race ==9 & sex ==1); lt_ss_m <- lt_ss_m[-c(1:3)]; lt_ss_m <- as.matrix(lt_ss_m) |
| assign(paste("race", 9, sep=""), transrate(lt_ss_m, lt_ss_f, yearlim=c(1970,2015), int.length=1)) |
|  |
| # install.packages("relsurv") |
| library(relsurv) |
| myratetable <- joinrate(list(race1=race1, race2=race2, race3=race3, race7=race7, race9=race9), dim.name="race") |
| class(myratetable) |
|  |
| # You can also use survexp.usr, survexp.us, survexp.mn, or slopop as a rate table... |
| # ?survexp.usr |
